# Analysis of Phylogenetic Variation of *Stenotrophomonas maltophilia* Reveals Human-Specific Branches

**DOI:** 10.3389/fmicb.2018.00806

**Published:** 2018-04-26

**Authors:** Joerg Steinmann, Uwe Mamat, Ebrahim M. Abda, Lisa Kirchhoff, Wolfgang R. Streit, Ulrich E. Schaible, Stefan Niemann, Thomas A. Kohl

**Affiliations:** ^1^Institute of Medical Microbiology, University Hospital Essen, University of Duisburg-Essen, Essen, Germany; ^2^Institute of Clinical Hygiene, Medical Microbiology and Infectiology, Paracelsus Medical University, Nuremberg, Germany; ^3^Cellular Microbiology, Priority Research Area Infections, Research Center Borstel, Borstel, Germany; ^4^Department of Microbiology and Biotechnology, Biocenter Klein Flottbek – University of Hamburg, Hamburg, Germany; ^5^TTU-TB, German Center for Infection Research, Borstel, Germany; ^6^Molecular and Experimental Mycobacteriology, Priority Research Area Infections, Research Center Borstel, Borstel, Germany

**Keywords:** *Stenotrophomonas maltophilia*, next generation sequencing, whole genome sequencing, biofilm, proteolytic activity, lipopolysaccharide, cystic fibrosis

## Abstract

*Stenotrophomonas maltophilia* is a non-fermenting Gram-negative bacterium that is ubiquitous in the environment. In humans, this opportunistic multi-drug-resistant pathogen is responsible for a plethora of healthcare-associated infections. Here, we utilized a whole genome sequencing (WGS)-based phylogenomic core single nucleotide polymorphism (SNP) approach to characterize *S. maltophilia* subgroups, their potential association with human infection, and to detect any possible transmission events. In total, 89 isolates (67 clinical and 22 environmental) from Germany were sequenced. Fully finished genomes of five strains were included in the dataset for the core SNP phylogenomic analysis. WGS data were compared with conventional genotyping results as well as with underlying disease, biofilm formation, protease activity, lipopolysaccharide (LPS) SDS–PAGE profiles, and serological specificity of an antibody raised against the surface-exposed O-antigen of strain *S. maltophilia* K279a. The WGS-based phylogenies grouped the strains into 12 clades, out of which 6 contained exclusively human and 3 exclusively environmental isolates. Biofilm formation and proteolytic activity did correlate neither with the phylogenetic tree, nor with the origin of isolates. In contrast, the genomic classification correlated well with the reactivity of the strains against the K279a O-specific antibody, as well as in part with the LPS profiles. Three clusters of clinical strains had a maximum distance of 25 distinct SNP positions, pointing to possible transmission events or acquisition from the same source. In conclusion, these findings indicate the presence of specific subgroups of *S. maltophilia* strains adapted to the human host.

## Introduction

*Stenotrophomonas maltophilia* is an opportunistic, non-fermentative, Gram-negative, and ubiquitous Gammaproteobacterium. This bacterium has environmental origins (water, soil), but is known to cause a diverse range of infections in humans (Denton and Kerr, 1998; Ryan et al., 2008; Brooke, 2012). It is usually considered as a pathogen with reduced virulence, but it can cause a broad spectrum of nosocomial infection complications with a considerable mortality rate of up to 37.5% (Falagas et al., 2009). The most common clinical manifestations caused by *S. maltophilia* include respiratory tract infections (Nseir et al., 2006; Nicodemo and Paez, 2007; Weber et al., 2007) and bacteremia (Lai et al., 2004; Chang et al., 2014).

*Stenotrophomonas maltophilia* exhibits high levels of intrinsic resistance to multiple classes of antibiotics (e.g., carbapenems and aminoglycosides). In addition, several mechanisms are able to contribute to acquired antimicrobial resistance (e.g., against fluorchinolones, polymyxins) resulting in a multi-drug resistance profile (Crossman et al., 2008; Ryan et al., 2009; Brooke, 2012). *S. maltophilia* is recognized by its ability to form biofilms on abiotic surfaces including glass and plastics like polystyrene (Brooke, 2012; Vidigal et al., 2014), as well as on host tissues such as bronchial epithelial cells (Pompilio et al., 2010). These characteristics make *S. maltophilia* an emerging global multi-drug-resistant opportunistic pathogen (Brooke et al., 2017).

In addition to nosocomial infections in hospitalized patients, *S. maltophilia* is able to chronically colonize the airways of CF patients in a manner similar to *Pseudomonas aeruginosa* or *Staphylococcus aureus*. It was shown that chronic *S. maltophilia* infection is an independent risk factor for pulmonary exacerbation in CF patients, associated with decline in FEV1 and a threefold increased risk for death or lung transplantation within the CF patient population (Waters et al., 2011, 2013; Vidigal et al., 2013).

Molecular typing studies uncovered a high degree of genetic diversity between most *S. maltophilia* strains from hospitalized individuals as well as between those from CF patients (Denton and Kerr, 1998; Valdezate et al., 2004; Gülmez and Hasçelik, 2005; Kaiser et al., 2009; Wu et al., 2011; Vidigal et al., 2014; Youenou et al., 2015). The molecular typing methods used so far include amplified fragment length polymorphism (AFLP), restriction fragment length polymorphism (RFLP), 16S rRNA sequencing, multi locus sequence typing (MLST), repetitive element palindromic (rep)-PCR, and *gyrB* sequencing, each producing varying results with no commonly accepted standard or defined nomenclature (Adamek et al., 2011).

In contrast to these classical typing techniques that only interrogate a small part of the genome, next generation sequencing technologies allow for the near complete analysis of a bacterial genome, i.e., whole genome sequencing (WGS). This method provides a single-base-pair resolution between isolates, making it the ultimate molecular typing method for bacteria (Snyder et al., 2013). Recent studies have demonstrated that analysis of bacterial genomes based on single nucleotide polymorphisms (SNPs) enables the definition of relatedness between epidemiologically associated isolates and tracking of bacterial evolution over time, including in long-term *S. maltophilia* colonization of CF patients (Jelsbak et al., 2007; Bragonzi et al., 2009; Lewis, 2010; Eppinger et al., 2011; Chung et al., 2017; Esposito et al., 2017). A very recent study found that the bacterial adaptation of *S. maltophilia* to the CF lung is associated with consistent genotypic and phenotypic heterogeneity (Esposito et al., 2017). Here, we performed WGS based phylogenomic analyses of a diverse collection of human isolates (mainly from CF and intensive care unit patients), and 22 environmental isolates. The collection represents the known diversity of *S. maltophilia* and *Stenotrophomonas rhizophila*, including representatives of phylogenetic groups previously defined by traditional genotyping methods (Adamek et al., 2011). The phylogenomic classifications have been linked with underlying diseases, phenotypical assays for biofilm formation, extracellular proteolytic activity and lipopolysaccharide (LPS) pattern characterization.

## Materials and Methods

### Strain Collection

In total, we analyzed 89 *S. maltophilia* and *S. rhizophila* strains, including 58 isolates collected by different institutes, as well as 31 strains chosen from a recent publication, which compared traditional genotyping methods, i.e., rep-PCR, *gyrB* sequencing, MLST, AFLP, and 16S rRNA sequencing, as respective group representatives (Adamek et al., 2011). Altogether, 67 (75%) strains in the collection were isolated from human samples and 22 (25%) from environmental sources. For the phylogenetic analysis, we also included the data from five fully sequenced genomes of *S. maltophilia* strains, namely K279a (NC_010943.1), D457 (NC_017671.1), JV3 (NC_015947.1), R551-3 (NC_011071.1), and *S. rhizophila* DSM14405 (CP007597.1). These strains were included as reference and control strains (Supplementary Table S1).

The 58 isolates were collected and provided by various institutes in Germany. One human isolate was provided by the University Hospital Muenster (Barbara Kahl), 41 human isolates and 8 environmental isolates by the University Hospital Essen (JS), 7 environmental strains were from the University of Hamburg (WS), and 22 human and 6 environmental isolates were provided by the Karlsruhe Institute of Technology (Adamek et al., 2011). In addition, one human isolate was provided by the Belgian Co-ordinated Collections of Microorganisms (LMG11112), one human and one environmental isolate by the Leibniz Institute DSMZ – German Collection of Microorganisms and Cell Cultures (DSM-50170 and DSM-14405), and one human isolate by Wolfgang Streit (K279a), originally obtained from the Avison (2000) laboratory.

### Bacterial Strains and Growth Conditions

All *S. maltophilia* and *S. rhizophila* strains used in the present study are listed in Supplemental Table S1. The strains were grown at 37°C or 30°C in either lysogeny broth (LB) or Brain Heart Infusion media. *Escherichia coli* strains SY327 [Δ(*lac pro*) *argE*(*Am*) *recA56 rif^R^ nalA*λ *pir*] (Miller and Mekalanos, 1988) and DH5α [F^-^ Φ80*lacZ*ΔM15 Δ(*lacZYA-argF*) U169 *recA1 endA1 hsdR17*(rK^-^mK^+^) *phoA supE44 thi-1 gyrA96 relA1*λ^-^] were routinely cultured at 37°C in LB medium. Chloramphenicol at concentrations of 30 and 60 μg/ml, kanamycin (30 μg/ml), tetracycline (50 μg/ml), or norfloxacin (5 μg/ml) were added to the media as required.

### Genomic DNA Isolation

The RNA-free genomic DNA of *S. maltophilia* and *S. rhizophila* strains was isolated from 1-ml overnight cultures using the DNeasy Blood & Tissue Kit according to the recommendations of the manufacturer (Qiagen, Hilden, Germany).

### Whole Genome Sequencing and Data Analysis

From extracted genomic DNA, sequencing libraries were constructed with the Nextera XT kit and run on the HiSeq (2 × 150 bp) or MiSeq (2 × 250 bp or 2 × 300 bp) sequencing platforms (Illumina, San Diego, CA, United States). Reads were mapped to the genome of *S. maltophilia* K279a (GenBank ID: NC_010943.1) with the alignment program BWA, and the mappings were refined with the GATK and Samtools toolkits. For a variant detection in the mapped reads, we employed Samtools and Perl scripts to filter for minimum thresholds of at least four reads in both forward and reverse orientation indicating an allele with a Phred score of at least 20 and an allele frequency above 75%. For phylogenetic analysis, variant positions were combined, supplementing the joint list with the respective information from the original mappings where necessary. SNP positions with a clear base call in all isolates and fulfilling our thresholds in at least 95% of the isolates were concatenated to a sequence alignment. From this, we calculated a maximum-likelihood tree with FastTree (Price et al., 2010) with a general time reversible substitution model, 1,000 resamples and Gamma20-likelihood optimization to account for the rate of heterogeneity among sites. The consensus tree was rooted with the “midpoint root” option in FigTree, and nodes were arranged in increasing order. We built a NeighborNet splitstree using the program Splitstree4 (Huson and Bryant, 2006) with default settings. Based on the set of SNP positions, isolates were also grouped together by a maximum distance of distinct SNP positions to the nearest group member.

For a genome-wide alignment of fully finished genomes of *S. maltophilia* strains K279a (NC_010943.1), D457 (NC_017671.1), JV3 (NC_015947.1), R551-3 (NC_011071.1), and *S. rhizophila* DSM14405 (CP007597.1), we employed Mauve using the progressive algorithm with default settings (Darling et al., 2004) (Supplementary Figure S1).

### Biofilm Formation Assay

Qualitative assessment of biofilm formation among *S. maltophilia* and *S. rhizophila* isolates was performed according to O’Toole (2011) with the following minor modifications. The microtiter plates (96-well plates, round-bottom, Sarstedt, Nürmbrecht, Germany) were pretreated with acetone for 10 s to enhance biofilm adhesion. All *S. maltophilia* and *S. rhizophila* isolates were cultured overnight in 96-well microtiter plates in LB. Each isolate was diluted in fresh LB to achieve a cell density equivalent of 10^8^ CFU/ml. A total of 100 microliters of each diluted culture was transferred into the wells and incubated at 37°C (human isolates) or 30°C (environmental isolates) for 24 h. Culture supernatants were discarded, and plates were allowed to dry at 60°C for 1 h. Quantification of biofilms was performed by staining the samples with a 2% solution of crystal violet and subsequent solubilization with 30% acetic acid. The crystal violet solution (125 μl) was added to each well, incubated at room temperature (RT) for 15 min and carefully washed three times with water to remove excess dye. The plate was dried at RT overnight. Finally, the dye was dissolved with 125 μl of 30% acetic acid for 15 min and transferred to a flat bottom microtiter plate. The absorbance of solubilized crystal violet was measured at OD_550_ using 30% acetic acid as a reference.

### Protease Assay

Extracellular proteolytic activity was assessed using red-fluorescent BODIPY TR-X casein from the EnzChek Protease Assay Kit as a substrate in accordance with the specifications of the manufacturer (Molecular Probes, Eugene, OR, United States). Overnight cultures of *S. maltophilia* and *S. rhizophila* strains were diluted with fresh medium to an OD_600_ of 0.05 and then allowed to continue growth under aerobic conditions with agitation (200 rpm) for 6 h. At this point, the cultures were adjusted again with fresh medium to an OD_600_ of 0.05 and grown for an additional 16 h, followed by measurements of the optical densities of the suspensions at 600 nm and sedimentation of the cells by centrifugation. The assay for secreted proteolytic activity was performed in a total volume of 200 μl with 10 μl of culture supernatants in white 96-well Nunc MicroWell polystyrene plates with cell culture surface coating (Thermo Fisher Scientific, Schwerte, Germany). The change in fluorescence was measured at 37°C for a period of 110 min using the GloMax-Multi+ Detection System (Promega, Mannheim, Germany). The basal fluorescence level of the negative control was subtracted from the fluorescence measured for each sample. Protease activity is expressed as fluorescence change in the culture supernatant of a bacterial culture with an OD_600_ of 1.0.

### Construction of an Unmarked *S. maltophilia* K279a Δ*rmlBACD* Deletion Mutant

Standard recombinant DNA methods were used for nucleic acid preparation and analysis (Sambrook and Russell, 2001). KOD Hot Start DNA Polymerase (Novagen, Nottingham, United Kingdom), Wizard Plus SV Minipreps DNA Purification System (Promega), High Pure PCR Product Purification Kit (Roche, Mannheim, Germany), DNA Clean & Concentrator-5 Kit (Zymo Research, Tustin, CA, United States), as well as DNA restriction endonucleases, T4 DNA ligase and DreamTaq DNA Polymerase (Thermo Fisher Scientific) were used as advised by the manufacturers. To construct an *S. maltophilia* K279a mutant defective in biosynthesis of the O-side chain of the LPS, the entire *rmlBACD* (*smlt0647-smlt0650*) operon coding for synthesis of dTDP-rhamnose, the immediate precursor for rhamnose residues of the O-repeating units, was deleted. The mutagenesis method is based on the pGPI-SceI/pDAI-SceI-SacB system, which was originally developed for bacteria of the genus *Burkholderia* (Flannagan et al., 2008; Aubert et al., 2014) and recently used for the generation of unmarked deletion mutants of *S. maltophilia* (Abda et al., 2015). Briefly, for deletion of the genomic region containing the *rmlBACD* operon of *S. maltophilia* K279a, the knockout plasmid pUDK021 was constructed in *E. coli* SY327, a cloning host for plasmids containing the R6Kγ origin of replication. As a first step, the primer pair KOsmltrmlBACD1/KOsmltrmlBACD2 (**Table 1**) and genomic DNA of *S. maltophilia* K279a was used as a template to amplify a 733-bp fragment of the flanking region upstream of *rmlB* (*smlt0647*). The PCR product was digested with *Sph*I/*Kpn*I and cloned into the *Sph*I/*Kpn*I sites of pGPI-SceI-XCm, yielding plasmid pUDK020. The flanking region downstream of *rmlD* (*smlt0650*) of 725 bp was then obtained by PCR from the genomic DNA of *S. maltophilia* K279a with primers KOsmltrmlBACD3 and KOsmltrmlBACD4 (**Table 1**), followed by digestion of the PCR product with *Kpn*I/*Xba*I and cloning into the *Kpn*I/*Xba*I sites of pUDK020 to yield pUDK021. Successful construction of the plasmids was verified by DNA sequence analysis of the inserts. Using *E. coli* DH5α carrying the plasmid pRK2013 (Figurski and Helinski, 1979) as a helper strain and *E. coli* SY327/pUDK021 as the donor strain, the deletion plasmid pUDK021 was transferred to *S. maltophilia* K279a by triparental mating as described previously (Aubert et al., 2014; Abda et al., 2015). The K279a co-integrants were selected at 37°C on LB agar plates containing 60 μg/ml chloramphenicol and 5 μg/ml norfloxacin to counter-select against the *E. coli* helper and donor strains. Due to the activity of the pyrocatechol 2,3-dioxygenase encoded by the *xylE* reporter gene of the deletion plasmid, the development of a bright yellow color after spraying the biomass with 0.45 M pyrocatechol confirmed the integration of pUDK021 into the genome of co-integrants. The helper plasmid pDAI-SceI-SacB was then introduced into the K279a co-integrants by triparental mating with DH5α/pRK2013 and DH5α/pDAI-SceI-SacB as helper and donor strains, respectively. The selection for exconjugants was performed at 30°C on LB agar plates containing 50 μg/ml tetracycline and 5 μg/ml norfloxacin, followed by screening for excision of the deletion plasmids, i.e., the inability of pyrocatechol to turn the color of the exconjugants bright yellow and sensitivity of the strains to 60 μg/ml chloramphenicol. Finally, the plasmid pDAI-SceI-SacB was cured by sucrose counter-selection as described (Aubert et al., 2014). The deleted region of the resulting *S. maltophilia* K279a Δ*rmlBACD* mutant was verified by PCR and DNA sequence analysis (data not shown).

**Table 1:** Primers used in this study.

Primer	Sequence
KOsmltrmlBACD1	ATATTgcatgcATCTCGAGCTTGCTGGCGAA^a^
KOsmltrmlBACD2	CTAGACggtaccGCACTACTTGTTCTCCTGATCGAAATTC^b^
KOsmltrmlBACD3	CATGCggtaccAGGATTTTGGCATCGTGCTGC^b^
KOsmltrmlBACD4	CTTCTtctagaATGGCAACGATGCTGGACAG^c^


*^*a*^*SphI* site is shown in lower case letters. ^*b*^*Kpn*I site is shown in lower case letters.^*c*^*Xba*I site is shown in lower case letters.*

### LPS SDS–PAGE and Immunoblotting

The LPS preparations from proteinase K-digested whole-cell lysates of *S. maltophilia* and *S. rhizophila* isolates were separated on 12% sodium dodecyl sulfate polyacrylamide gel electrophoresis (SDS–PAGE) gels and stained with silver nitrate according to the method of Hitchcock and Brown (1983). The lysates were prepared from the biomass of overnight cultures grown on agar plates. For immunoblots, a rabbit polyclonal antibody against heat-inactivated *S. maltophilia* K279a cells was produced by Eurogentec, Seraing, Belgium. In order to use the antiserum for detection of homologous O-serotypes, the immune serum was adsorbed with heat-killed cells of the herein constructed *S. maltophilia* K279a Δ*rmlBACD* mutant as previously described (Schlecht and Schmidt, 1972). The LPS samples were electrotransferred from SDS–PAGE gels onto polyvinylidene difluoride (PVDF) membranes (Millipore, Merck, Darmstadt, Germany), followed by incubation of the blots with adsorbed anti-K279a antibody. The immunoblots were then treated with alkaline phosphatase-conjugated AffiniPure goat anti-rabbit immunoglobulin (Ig)G (H+L) (Dianova, Hamburg, Germany) and developed in the presence of nitroblue tetrazolium and 5-bromo-4-chloro-3-indolylphosphate substrate (Promega).

## Results

In total, 89 *S. maltophilia* and *S. rhizophila* isolates were sequenced successfully, with at least 240 Mbp sequence data generated per sample. Mapping to the K279a genome resulted in a mean coverage depth of 77.0-fold (range 35.6–222.4) of covered regions, and a mean coverage breadth of the reference sequence of 79.0% (range 41–100%). Fully finished genomes of five strains were added as controls and references K279a (NC_010943.1), D457 (NC_017671.1), JV3 (NC_015947.1), R551-3 (NC_011071.1), and *S. rhizophila* DSM14405 (CP007597.1). For the full analysis of all 94 datasets of this study, 1,928,889 bp out of the 4,851,126 bp of the K279a reference genome complied with the chosen thresholds and were used to derive a set of 408,860 SNP positions for the calculation of phylogenetic trees.

**FIGURE 1:**
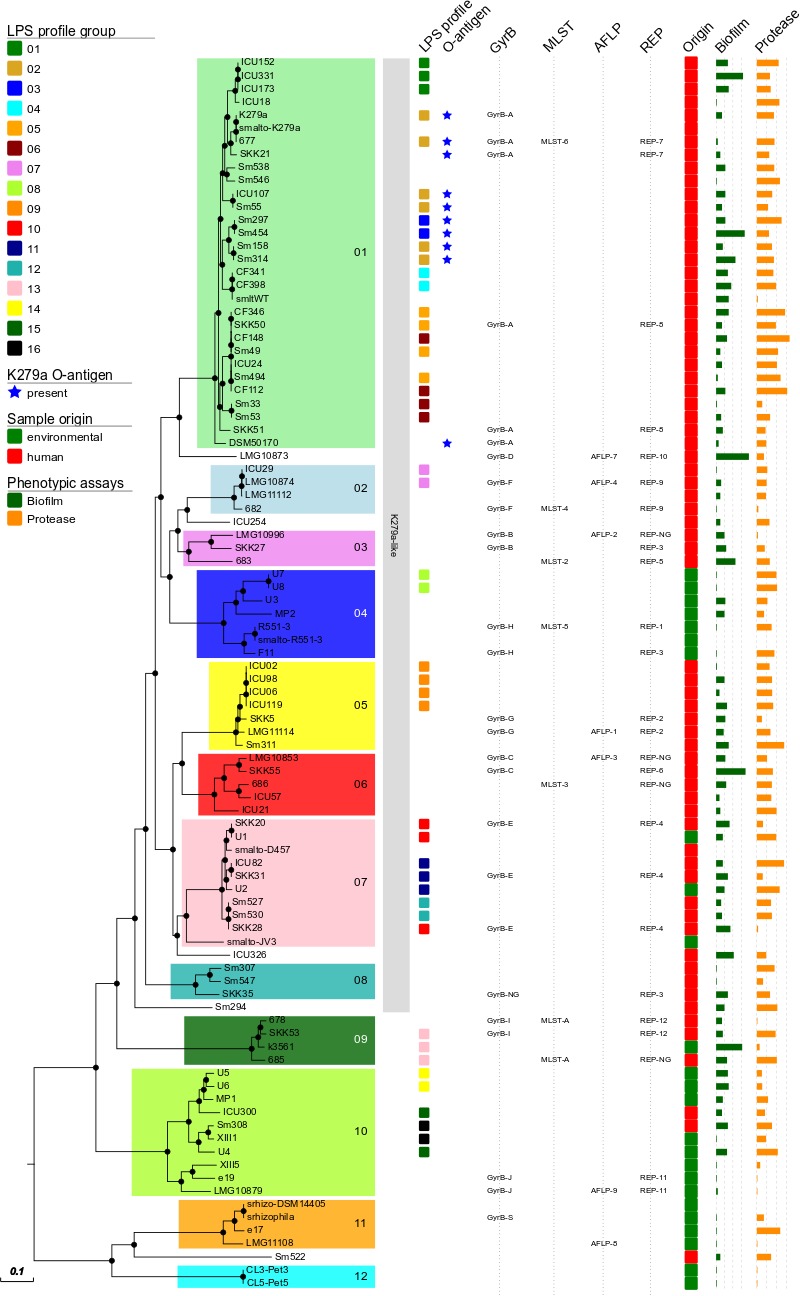
Maximum-likelihood tree calculated from the 408,860 aligned SNP positions of the 94 datasets of *S. maltophilia* and *S. rhizophila*. All but five strains can be classified into 12 major phylogenetic groups, designated groups 01–12. Groups of LPS profiles, presence of a K279a-like O-antigen, genotypes (GyrB, AFLP, rep-PCR, MLST) and origin of the isolate are indicated colored squares according to the legend on the left. On the right, the results of the biofilm (green bars) and protease (orange bars) assays are given as relative activities. Proteolytic activity is depicted as relative fluorescence/OD600 unit (110-min values) and values below 0 were set to 1. For the biofilm assay, the absorbance at 595 nm is depicted. Node resampling support of more than 90% is indicated by black dots. K279a-like: 75% of the K279a genome sequence could be recovered from WGS data. Black dots on nodes indicate a resampling support greater 90%.

**FIGURE 2:**
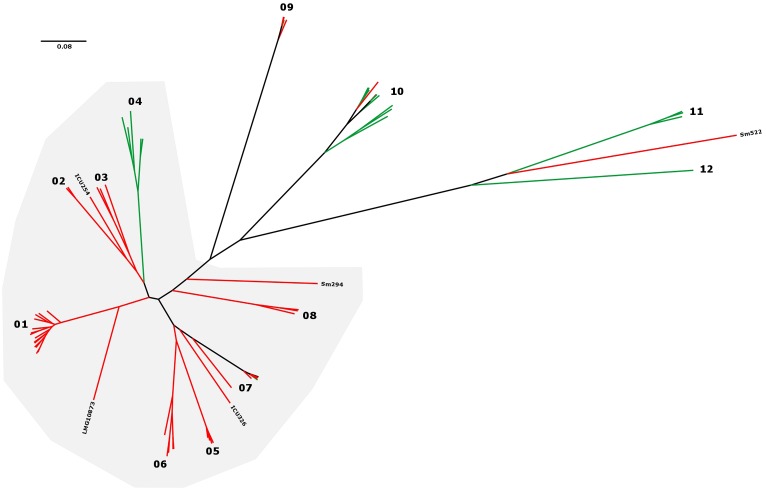
*S. maltophilia* and *S. rhizophila* maximum-likelihood tree in radial tree layout. The 12 phylogenetic groups are indicated by numbers, and isolate origin mapped on the tree in green (environmental) and red (human). K279a-like strains for which 75% of the K279a genome sequence could be recovered from WGS data are indicated by a gray background.

Group definition with a threshold of a maximum distance of 50.000 distinct SNP positions to the nearest group member resulted in 12 groups/clades in the *S. maltophilia* and *S. rhizophila* phylogeny (designated groups 01–12), which correspond well with GyrB, AFLP, rep-PCR (with the notable exception of rep-3), and MLST typing results (**Figure 1** and **Table 2**). The 12 groups are separated by large SNP differences in line with independent environmental acquisition (**Figure 2**). While both, a genome-wide genome alignment comparison of the five fully completed genomes (*S. maltophilia* K279a, D457, JV3, R551-3, and *S. rhizophila* DSM14405) and a NeighborNet splitstree built from the detected SNP positions of the 94 strains, indicate some genomic rearrangement, the distinction into separate groups remains stable in the NeighborNet tree (Supplementary Figure S2). Strikingly, the phylogenetic groups we defined almost exclusively comprise either isolates from the environment or human. Indeed, 9 out of the 12 groups were comprised entirely of human or environmental isolates, and the remaining three groups contained at least 75% either human or environmental isolates (**Table 2**).

**Table 2:** Defined groups of *S. maltophilia* and *S. rhizophila* isolates.

GROUP	HUMAN	ENVIR.	PERC. HUMAN (%)	PERC. ENVIR. (%)	GYRB	MLST	AFLP	REP-PCR	PROTEASE	BIOFILM
01	29	0	100	0	A	6	n.a.	7,8	657.3	1.07
02	4	0	100	0	F	4	4	9	377.9	0.51
03	3	0	100	0	B	2	2	3,5^NG^	175.6	1.09
04	0	6	0	100	H	5	n.a.	1,3	621.9	1.06
05	7	0	100	0	G	n.a.	1	2	586.1	1.03
06	5	0	100	0	C	3	3	6^NG^	587.0	0.81
07	6	2	75	25	E	n.a.	n.a.	4	563.1	0.93
08	3	0	100	0	NG	n.a.	n.a.	3	508.6	1.42
09	3	1	75	25	I	A	n.a.	12^NG^	414.8	1.90
10	2	8	20	80	J	n.a.	9	11	315.8	1.26
11	0	3	0	100	S	n.a.	8	n.a.	585.6	n.a.
12	0	2	0	100	n.a.	n.a.	n.a.	n.a.	n.a.	n.a.
UNGR.	5	0	100	0	*–*	*–*	*–*	*–*	*–*	*–*
TOTAL	67	22	75	25	*–*	*–*	*–*	*–*	*–*	*–*


*For each group derived from phylogenetic analysis, the number of human and environmental isolates is given, both in total number and as proportion (without NCBI datasets). Traditional typing results for group isolates are indicated, as well as the median value of the protease and biofilm assays. n.a. = not available; NG = non-grouped; UNGR = ungrouped; ENVIR = Environment; Perc. = Percentage.*

In addition to a clear separation of human and environmental isolates in most groups, the overall majority of human derived strains could be grouped close to the K279a branch (K279a-like) in the *S. maltophilia* and *S. rhizophila* phylogeny (**Figures 1**, **2**). Notably, neither proteolytic activity levels nor capabilities to form biofilms on polystyrene correlated with the phylogenetic separation (**Figure 1** and **Table 1**).

To get a first indication on group-specific pathobiological characteristics, we mapped biofilm and protease production on the tree (**Figure 1**). In general, the majority of the isolates formed biofilms on a plastic surface in microtiter plates. Only 19 strains failed to form detectable biofilms. Surprisingly, a large fraction of the environmental isolates [11 (50%)] did not form biofilms. This is in contrast to the clinical isolates, from which only 8 strains (12.5%) failed to form biofilms under the same experimental conditions. With respect to protease secretion, we noticed that only 9 isolates failed to produce significant amounts of protease, but no correlation with respect to the SNP analysis or their origin was detected.

**FIGURE 3:**
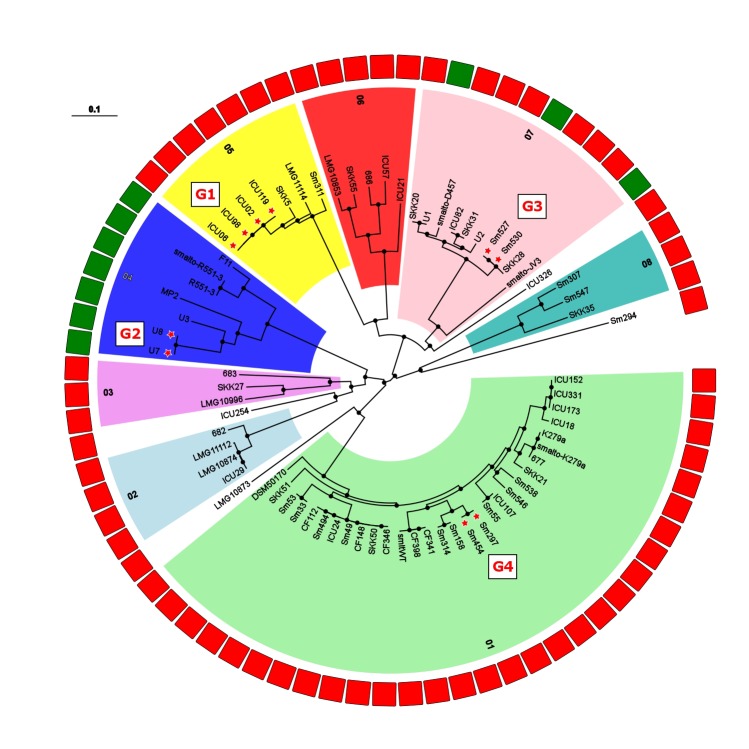
Maximum-likelihood tree built from 649,312 SNP positions of the set of 73 *S. maltophilia* strains marked as K279a-like in **Figure 1**. Phylogenetic groups are indicated by numbers, and isolate origin shown as rectangles in the outer ring in green (environmental) and red (human). Excluding datasets from full genomes, 4 groups of strains were detected with a threshold of 25 distinct SNP positions [threshold used for *M. abscessus* by Bryant et al. (2013)] and are indicated by red stars next to isolate names and labeled G1 (ICU02, ICU06, ICU98, ICU119), G2 (U7, U8), G3 (SM527, SM530), and G4 (SM297, SM454). Black dots on nodes indicate a resampling support greater 90%.

To obtain a deeper understanding of subgroup clonality, we performed a targeted analysis of 73 strains from which at least 75% of the K279a genome sequence could be recovered. This allows for an increased resolution of the resulting SNP phylogenies with 649,312 SNP positions detected and 3,061,984 bp out of the 4,851,126 bp reference sequence taken into account for the comparison (**Figure 3**). Overall, the isolates are separated by large SNP distances, reflecting their environmental occurrence. However, excluding full genome datasets, we identified four groups of strains for which group members were at a maximum distance of 25 distinct SNP positions from each other, possibly indicating transmission between patients: G1 (ICU02, ICU06, ICU98, ICU119), G2 (U7, U8), G3 (Sm527, Sm530), and G4 (Sm297, Sm454). In the cases of the ICU strain cluster (G1) and the CF groups (G3, G4), a spatio-temporal association was present, indicating that these patients were infected by the same strains presumably based on patient-to-patient or environment-to-patient transmission.

The LPS profiles of *S. maltophilia* and *S. rhizophila* were compared to identify identical ladder-like LPS patterns (Supplementary Figure S3). In spite of a significant heterogeneity of the LPS banding patterns, we have been able to assign 46 strains to a total of 16 LPS groups on the basis of electrophoretic mobility of the main bands, with at least two identical profiles within a group. Isolates with apparent unique LPS patterns or rough-type LPS lacking O-repeating units were ignored. Furthermore, the *S. maltophilia* and *S. rhizophila* strains were screened with a polyclonal antiserum against the O-antigen of *S. maltophilia* K279a. In order to obtain an antibody specifically directed against the O-antigenic chain of *S. maltophilia* K279a, a Δ*rmlBACD* deletion mutant of the strain lacking the gene cluster for dTDP-rhamnose synthesis was constructed. The polyclonal antibody against heat-inactivated *S. maltophilia* K279a wild-type cells was then adsorbed with heat-killed cells of the Δ*rmlBACD* mutant. Both the loss of O-substituted LPS in *S. maltophilia* K279a Δ*rmlBACD* and the O-specificity of the adsorbed immune serum was confirmed by SDS–PAGE and immunoblot analysis (Supplementary Figure S4). Immunoblot analyses of *Stenotrophomonas* spp. indicated serological cross-reactivity of the O-specific K279a antiserum with the O-antigens of 10.6% (10, including *S. maltophilia* K279a) of all isolates and 13.7% of isolates herein assigned to the group of 73 K279a-like strains, which suggested that these isolates expressed a structurally similar or even identical O-specific polysaccharide (Supplementary Figure S4). The serological cross-reactivity of the members of LPS-profile groups 02 and 03 was in good agreement with the phylogenetic tree (**Figure 1**), for which positive detection was restricted to clustered isolates closely related to K279a of LPS group 02, with the sole exception of strain DSM50170 displaying a unique LPS banding pattern. We also found that each group of K279a-like strains with a maximum distance of only 25 distinct SNP positions could be classified into separate LPS groups, each with an identical banding pattern to derive LPS groups 09 (ICU02, ICU06, ICU98, ICU119), 08 (U7, U8), 12 (Sm527, Sm530) and 03 (Sm297, Sm454), with the LPS of Sm297 and Sm454 showing, in addition, serological cross-reactivity with the O-specific K279a antibody.

## Discussion

*Stenotrophomonas maltophilia* is becoming increasingly relevant as a nosocomial pathogen. We used WGS to dissect the phylogenetic relationship of a set of 89 *S. maltophilia* and *S. rhizophila* isolates from human and environmental sources. Our phylogenetic analysis revealed a highly diverse population with deep branches separating groups of strains. Notably, these groups correspond well to those identified by traditional genotyping methods. Despite a generally conserved genome structure (Supplementary Figure S1), groups were distinguished by thousands of distinct SNP positions, with a reduced diversity within a “supergroup” of most human-derived K279a-like isolates (**Figure 1**). Interestingly, 9 out of the 12 groups consisted exclusively of either human (6 groups) or environmental isolates (3 groups). Out of the three remaining groups, two reflected the total ratio (75% human to 25% environmental isolates), and one comprised mostly environmental strains (80%). These data are in line with a previous MLST study from Germany which also found overrepresentation of some genogroups among human and environmental strains (Kaiser et al., 2009). Taken together, these results suggest a correlation between association with human diseases and genetic background. The identified groups may also differ with regard to their pathogenic potential, e.g., a higher pathogenic potential in strains closely related to the group defined by reference strain K279a. Alternatively, the strains within the very same lineage might have a higher potential to colonize humans and cause infectious diseases in comparison with others. The K279a reference strain also used in other studies was originally isolated from a septic patient’s blood sample (Crossman et al., 2008).

In contrast to our findings, Youenou et al. (2015) detected that genomic data from environmental and clinical *S. maltophilia* isolates did correlate neither to their origin nor to their antibiotic resistance profile. However, this study was limited to a small number of isolates (*n* = 14 in total; *n* = 5 from clinical origin). Another recent study also addressed the question of specific phylogenetic branches (Lira et al., 2017). A total of 10 environmental and 10 clinical isolates were sequenced and a large core genome with similar distributions of genes including virulence determinants was found. No specific evolutionary branches were detected. It was postulated that the evolution of *S. maltophilia* is based on the strain-specific acquisition of genes during adaptation to different microniches (Lira et al., 2017). Chung et al. (2017) revealed that, even within the lungs of one individual CF patient, the *S. maltophilia* isolates separated into several lineages with distinct phenotypes distinguished by adaptive mutations (Chung et al., 2017). This study also showed that mutations within certain strains were associated with specific pulmonary locations indicating selection driven by the micro-environment.

Another recent study investigated 91 *S. maltophilia* isolates from 10 CF patients over a period of 12 years using genotypic and phenotypic methods (Esposito et al., 2017). SNP-based and pangenome analysis showed three major lineages whereas phenotypic results (growth rate, biofilm formation, motility, mutation frequency, antibiotic susceptibility, and *in vivo* virulence) did not correlate with genomic data.

While our findings confirm a large genome diversity among environmental and human *S. maltophilia* isolates, their capacity to colonize CF-patients appears to be higher for certain defined groups. Furthermore, at least in few instances, we detected likely events of nosocomial transmission or acquisition from the same origin as already observed for *Mycobacterium abscessus* (Bryant et al., 2016) This warrants further investigations into transmission of *S. maltophilia* in the community and hospital setting to determine scale and universality of this phenomenon, as well as its importance for disease severity. Furthermore, it is tempting to speculate that the unequal distribution of human isolates in the *S. maltophilia* phylogeny is based on special virulence features of strains from groups associated with human infection.

One potential virulence factor is the O-specific polysaccharide that is the most variable part of the LPS molecule and determines the serological specificity of LPS and Gram-negative bacteria. It has further long been recognized that the O-polysaccharide is a fairly stable phenotypic marker that can be used to distinguish between groups of strains within a given species (Rimler, 1990; Mills et al., 1992; Aucken and Pitt, 1993; Jürgens and Fehrenbach, 1997; Santamaría et al., 1998). The enormous variability of O-polysaccharide structures has been attributed to evolutionary mechanisms that enabled alterations of the most exposed surface structure to maintain advantageous adaptations to particular environmental niches or evade niche-specific selection pressures or escape-specific antibody-based immunity. In fact, the structural diversity of O-polysaccharides reflects an extensive polymorphism at the genetic level, including gene mutations, genetic exchange events between different microorganisms, as well as recombination processes (Cunneen and Reeves, 2011). Varying numbers of repeating units in the O-specific polysaccharide result in unique ladder-like banding patterns of LPS separated by SDS–PAGE. Thereby, comparative LPS profiling and O-serotyping are valuable tools for epidemiological studies with high discriminatory power (Aucken and Pitt, 1993). Here, we show that cross-reactivity of an antiserum raised against the O-antigen of K279a correlates well with our phylogenetic analysis, with only two subgroups of closely related strains expressing the K279a-like O-side chain antigen, apart from one isolate, DSM50170, with a distinctly different gel profile.

However, the serological cross-reaction with DSM50170 indicates that the O-specific polysaccharide of K279a and reactive K279a-like isolates is similar to the structure of the O8-antigen of DSM50170, which consists of a branched tetra-saccharide with three rhamnopyranosyl residues in the main chain and 3-*O*-methylxylose as the substituent (Neal and Wilkinson, 1982; Knirel, 2011). Of note, a total of 31 different *S. maltophilia* O-serotypes and 16 O-antigenic structures have been described thus far, showing that most of the O-antigen repeating units of the *S. maltophilia* LPS have been branched tri- and tetra-saccharides of rhamnose, fucose, xylose, and glucose (Ryan et al., 2009; Knirel, 2011). While the LPS-based approach aimed to complement our WGS data has identified a number of closely related isolates, we herein confirm previous results on the variability of O-repeating unit structures within the genus *Stenotrophomonas*, which in fact reflects the versatility of the bacterium to adapt to various environmental niches.

The capability to form biofilms as well as to secrete proteolytic enzymes differed between individual strains but failed to correlate with the distinct groups, and was also not particularly high in groups containing only human isolates. These results are in accordance with a previous study including CF isolates (Esposito et al., 2017). The distribution of both properties, present or absent, across all groups suggests that both phenotypic traits can be universally present in the species complex but are not relevant for the pathogenic potential of a strain. However, both biofilm formation and proteolytic activity can be of relevance for isolates living in the environment or the human host depending on the individual niches.

A previous study combining comparative genomics, transcriptomics and physiological approaches with *S. rhizophila* DSM14405, a plant-associated *S. maltophilia* strain, and the human *S. maltophilia* K279a found habitat-specific genes in human vs. environmental strains (Alavi et al., 2014). Therefore, a systematic comparison of the pangenome of the highly diverse *S. maltophilia* and *S. rhizophila* species complex would be interesting. A prerequisite and ideal tool for this comparison would be the construction of a whole genome MLST scheme. Thereby, potential virulence factors could also be identified, which likely correlate either with the human or with the plant host.

In this context, it should be noted that the strain collection may have introduced a systematic bias since the majority of the human isolates analyzed were obtained at one site (University Hospital Essen, 41 out of 67 isolates). Similarly, the number of environmental isolates tested in this study was rather small, with just 22 sequenced strains, compared to the number of clinical isolates. In summary, WGS provided a high-resolution phylogeny of the *S. maltophilia* and *S. rhizophila* species complex. Our results indicate the presence of branches of strains adapted to the human host, predominantly CF patients, with the potential of human-specific virulence and pathogenicity. The reason for increased virulence, however, remains elusive, and needs to be investigated in future studies.

## Availability of Data and Materials

The datasets supporting the conclusions of this article are available in the European Nucleotide Archive (ENA) repository of the European Molecular Biology Laboratory (EMBL). All accession numbers are stated in Supplementary Table S1.

## Ethics Statement

All clinical samples of the present study were analyzed after performing conventional microbiological diagnostics. The study did not include patient’s details and did not result in additional constraints for the patients. All data were anonymously analyzed without patients’ consent due to the retrospective nature of the study. All analyses were carried out in accordance with approved guidelines.

## Author Contributions

Study design: JS, US, SN, and TK. Carrying out experiments: UM, EA, WS, and TK. Writing: JS, UM, WS, and TK. Data analysis, final revision, and approval of final version: JS, UM, EA, LK, WS, US, SN, and TK.

## Conflict of Interest Statement

The authors declare that the research was conducted in the absence of any commercial or financial relationships that could be construed as a potential conflict of interest.

## Abbreviations

AFLP: amplified fragment length polymorphism
CF: cystic fibrosis
LB: lysogeny broth
LPS: lipopolysaccharide
MLST: multi-locus sequence type
NGS: next generation sequencing
RFLP: restriction fragment length polymorphism
RT: room temperature
SNP: single nucleotide polymorphisms
WGS: whole genome sequencing
