# Supplementary material for: Analysis of Phylogenetic Variation of Stenotrophomonas maltophilia Reveals Human-Specific Branches
Source: Front Microbiol. 2018 Apr 26;9:806. doi: 10.3389/fmicb.2018.00806 (PMC5932162; doi:10.3389/fmicb.2018.00806)
Supplement: Supplementary file 2 [file Image_1.PDF]

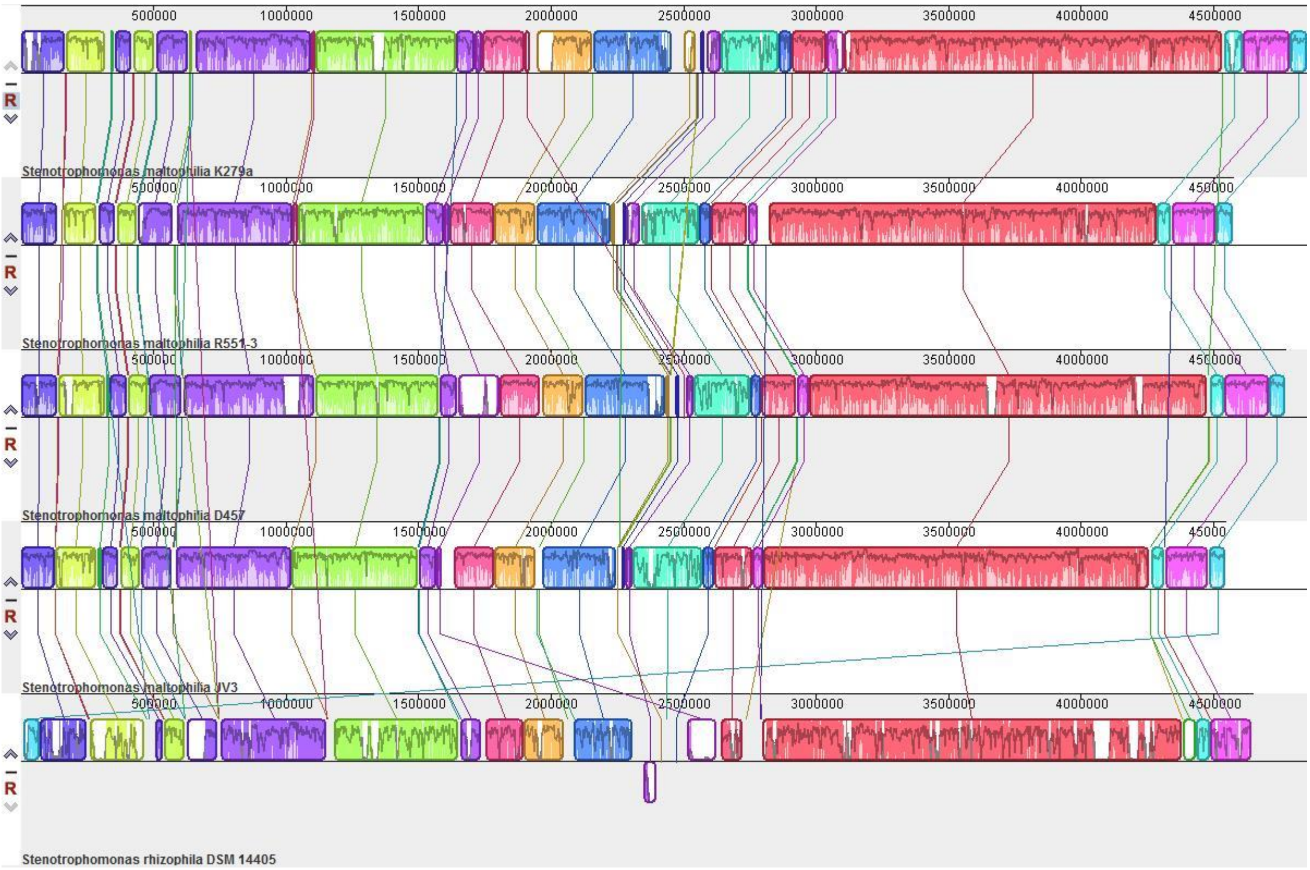


**Supplementary Fig S1.** Mauve Alignment of five fully finished *S. maltophilia* and *S. rhizophila* genomes: *S. maltophilia* strains K279a (NC_010943.1), D457 (NC_017671.1), JV3(NC_015947.1), R551-3 (NC_011071.1), and *S. rhizophila* DSM14405 (CP007597.1). The software was run with the progressive alignment option and used with default settings.
