# Supplementary material for: Analysis of Phylogenetic Variation of Stenotrophomonas maltophilia Reveals Human-Specific Branches
Source: Front Microbiol. 2018 Apr 26;9:806. doi: 10.3389/fmicb.2018.00806 (PMC5932162; doi:10.3389/fmicb.2018.00806)
Supplement: Supplementary file 3 [file Image_2.pdf]

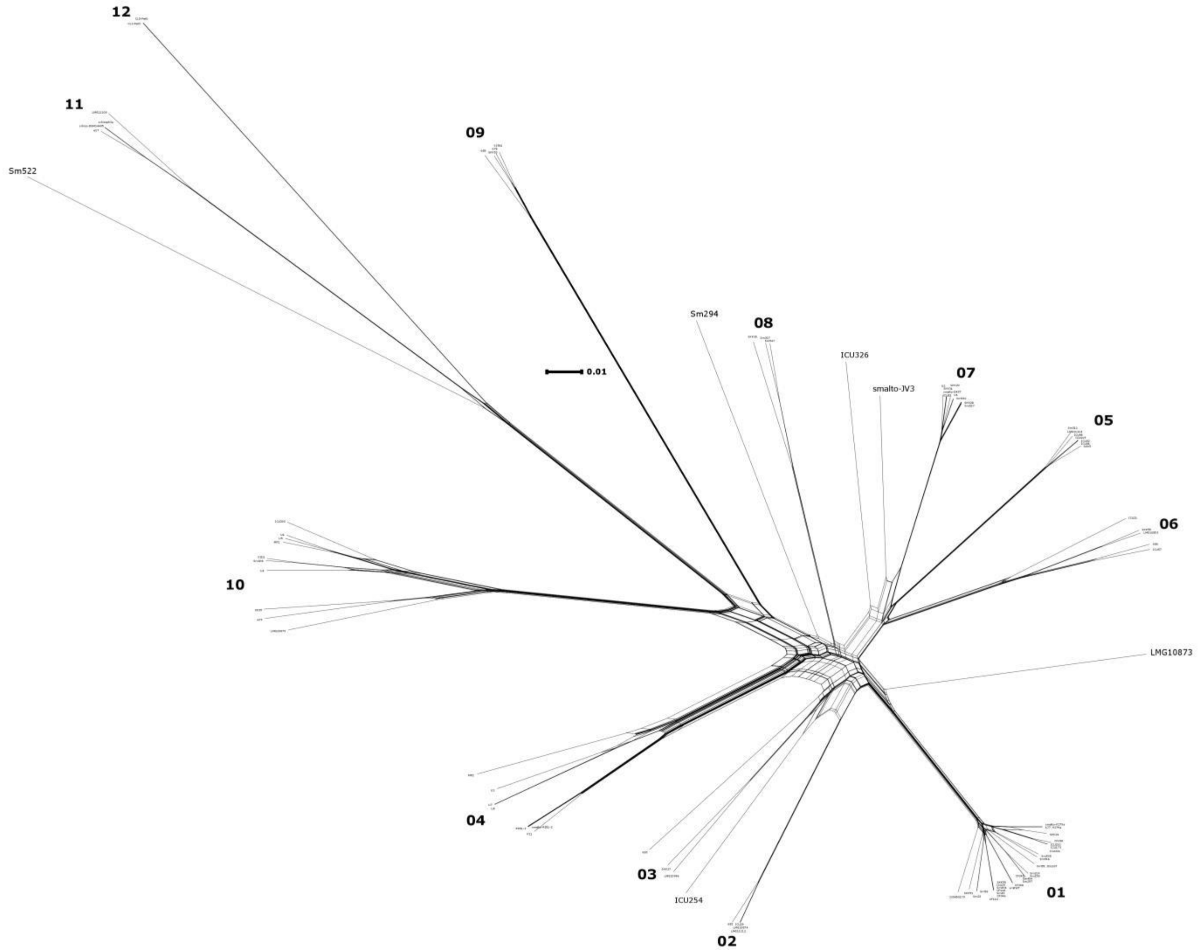

Supplementary Fig 2. NeighborNet splitstree built from the 408,860 aligned SNP positions of the 94 *S. maltophilia* and *S. rhizophila* datasets using the program Splitstree4 with default settings. Groups as shown in Figure 1 are indicated.
