# Supplementary material for: Analysis of Phylogenetic Variation of Stenotrophomonas maltophilia Reveals Human-Specific Branches
Source: Front Microbiol. 2018 Apr 26;9:806. doi: 10.3389/fmicb.2018.00806 (PMC5932162; doi:10.3389/fmicb.2018.00806)
Supplement: Supplementary file 4 [file Image_3.pdf]

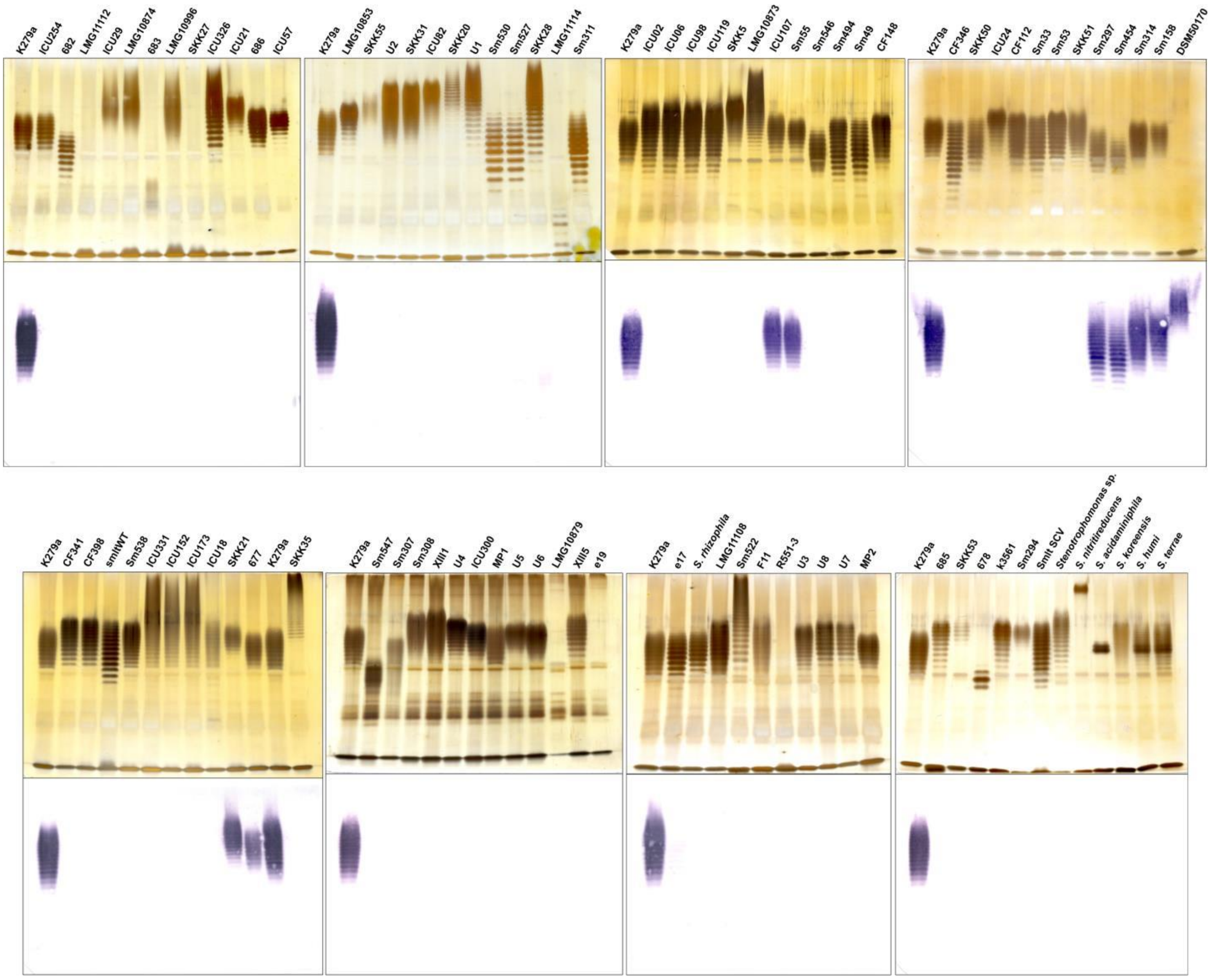

Supplementary Fig S3. LPS profile typing and O-serotyping of *S. maltophilia* and *S. rhizophila* isolates. LPS preparations from proteinase-K digested whole-cell lysates were separated on 12% SDS-PAGE gels and stained with silver nitrate (*top panels*). Corresponding immunoblots were developed with an antiserum specific for the O-polysaccharide of *S. maltophilia* K279a (*bottom panels*). The whole-cell lysate of K279a was used in each first lane as a control.
