# Supplementary material for: Analysis of Phylogenetic Variation of Stenotrophomonas maltophilia Reveals Human-Specific Branches
Source: Front Microbiol. 2018 Apr 26;9:806. doi: 10.3389/fmicb.2018.00806 (PMC5932162; doi:10.3389/fmicb.2018.00806)
Supplement: Supplementary file 5 [file Image_4.PDF]

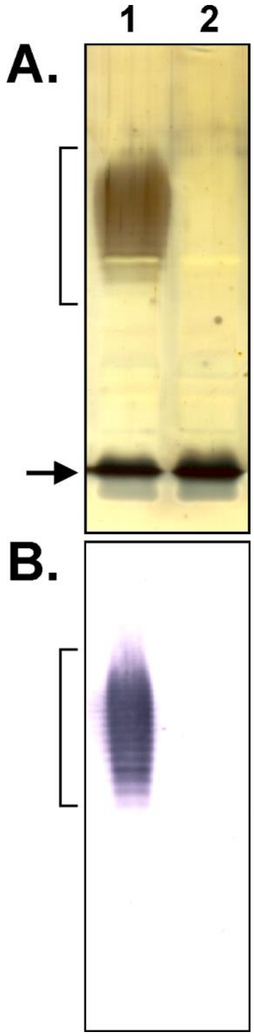

Supplementary Figure S4. SDS-PAGE and immunoblot analysis of the LPS profiles of *S. maltophilia* wild-type strain K279a (*lane 1*) and the K279a Δ*rmlBACD* deletion mutant (*lane 2*). Proteinase-K digested whole-cell lysates were separated on 12% SDS-PAGE gels and stained with silver nitrate (*A.*). The corresponding immunoblot was developed with an antiserum specific for the O-antigen of *S. maltophilia* K279a (*B.*). The *brackets* define the O-antigen repeat bands while the *arrow* denotes the unligated LPS core.
